# Genomics reveals repeated landlocking of diadromous fish on an isolated island

**DOI:** 10.1002/ece3.10987

**Published:** 2024-02-16

**Authors:** Motia G. Ara, Graham A. McCulloch, Ludovic Dutoit, Graham P. Wallis, Travis Ingram

**Affiliations:** ^1^ Department of Zoology University of Otago Dunedin New Zealand; ^2^ Department of Marine Fisheries and Oceanography Patuakhali Science and Technology University Patuakhali Bangladesh

**Keywords:** approximate Bayesian computation, Chatham Island, diadromy, genotyping‐by‐sequencing, New Zealand, Retropinnidae

## Abstract

Landlocking of diadromous fish in freshwater systems can have significant genomic consequences. For instance, the loss of the migratory life stage can dramatically reduce gene flow across populations, leading to increased genetic structuring and stronger effects of local adaptation. These genomic consequences have been well‐studied in some mainland systems, but the evolutionary impacts of landlocking in island ecosystems are largely unknown. In this study, we used a genotyping‐by‐sequencing (GBS) approach to examine the evolutionary history of landlocking in common smelt (*Retropinna retropinna*) on Chatham Island, a small isolated oceanic island 800 kilometres east of mainland New Zealand. We examined the relationship between Chatham Island and mainland smelt and used coalescent analyses to test the number and timing of landlocking events on Chatham Island. Our genomic analysis, based on 21,135 SNPs across 169 individuals, revealed that the Chatham Island smelt was genomically distinct from the mainland New Zealand fish, consistent with a single ancestral colonisation event of Chatham Island in the Pleistocene. Significant genetic structure was also evident within the Chatham Island smelt, with a diadromous Chatham Island smelt group, along with three geographically structured landlocked groups. Coalescent demographic analysis supported three independent landlocking events, with this loss of diadromy significantly pre‐dating human colonisation. Our results illustrate how landlocking of diadromous fish can occur repeatedly across a narrow spatial scale, and highlight a unique system to study the genomic basis of repeated adaptation.

## Graphical abstract

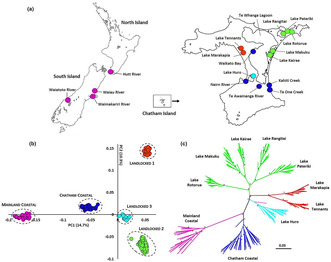

## 1 INTRODUCTION

Diadromy is a life history strategy that involves migrations between marine and freshwater environments (Delgado et al., 2019; Delgado & Ruzzante, 2020; McDowall, 2008). As the marine life stage provides an opportunity for ongoing gene flow in diadromous fish, there is often very limited genetic structuring among populations (Delgado et al., 2019; Waters et al., 2000). Diadromous fish can, however, become isolated in freshwater ecosystems when natural or anthropogenic barriers prevent their migration to the ocean (Lee & Bell, 1999; Waters et al., 2020). In facultatively diadromous species that can complete their life history in freshwater, this loss of migratory connectivity results in the formation of landlocked populations in lakes.

Landlocking can have a number of important consequences for normally diadromous fish. Landlocked fish populations often experience limited or no gene flow with other populations, resulting in the development of significant genetic differentiation among populations (Allibone & Wallis, 1993; Bowersox et al., 2016; Drevecky et al., 2013; Lemopoulos et al., 2018; Perrier et al., 2013; Salisbury et al., 2018, 2022; Tonteri et al., 2007; Waters & Wallis, 2001a, 2001b), which can in some cases lead to speciation (Allibone et al., 1996; Burridge et al., 2012). Isolated landlocked populations also often have reduced effective population size and increased levels of inbreeding, which may significantly increase extinction risk (Samad‐zada et al., 2021). Landlocked populations are typically exposed to a distinct selective environment compared to their diadromous counterparts, as they often experience different salinity, temperature, oxygen, pH and water velocities, and may encounter new prey, predators, parasites and symbionts (Lee et al., 2011). These environments may drive changes in physiological, behavioural, morphological and life history traits involved in swimming ability, predator or parasite resistance and feeding (Posavi et al., 2020; Velotta et al., 2018). Phenotypic shifts can occur through natural selection acting on standing genetic variation or new mutations, as well as phenotypic plasticity in response to environmental differences (Augspurger, 2017; Hicks et al., 2021; Jacobs et al., 2020; Jones et al., 2012; Oke et al., 2016).

The Pacific region, including Aotearoa New Zealand and its offshore islands, has a freshwater fish fauna dominated by diadromous species and their freshwater‐resident descendants (McDowall, 1990). A particularly common form of diadromy in this region is amphidromy which features freshwater spawning and a larval marine phase followed by a postlarval migration back to freshwater where the majority of growth occurs. A number of New Zealand's native amphidromous species are facultatively diadromous, including the common smelt, *Retropinna retropinna* (Kattel & Closs, 2007; Northcote et al., 1992; Northcote & Ward, 1985; Ward et al., 2005). Common smelt is widely distributed throughout New Zealand and has become landlocked in North and South Island lakes through natural colonisation as well as human translocation (McDowall, 1979; Rowe & Kusabs, 2007; Strickland, 1993). A number of landlocked populations of common smelt also occur on Chatham Island (Rēkohu), which is distinguished by its large number of low‐elevation dune lakes and by the absence of the introduced fish species that have impacted native species distributions throughout most of New Zealand.

**FIGURE 1:**
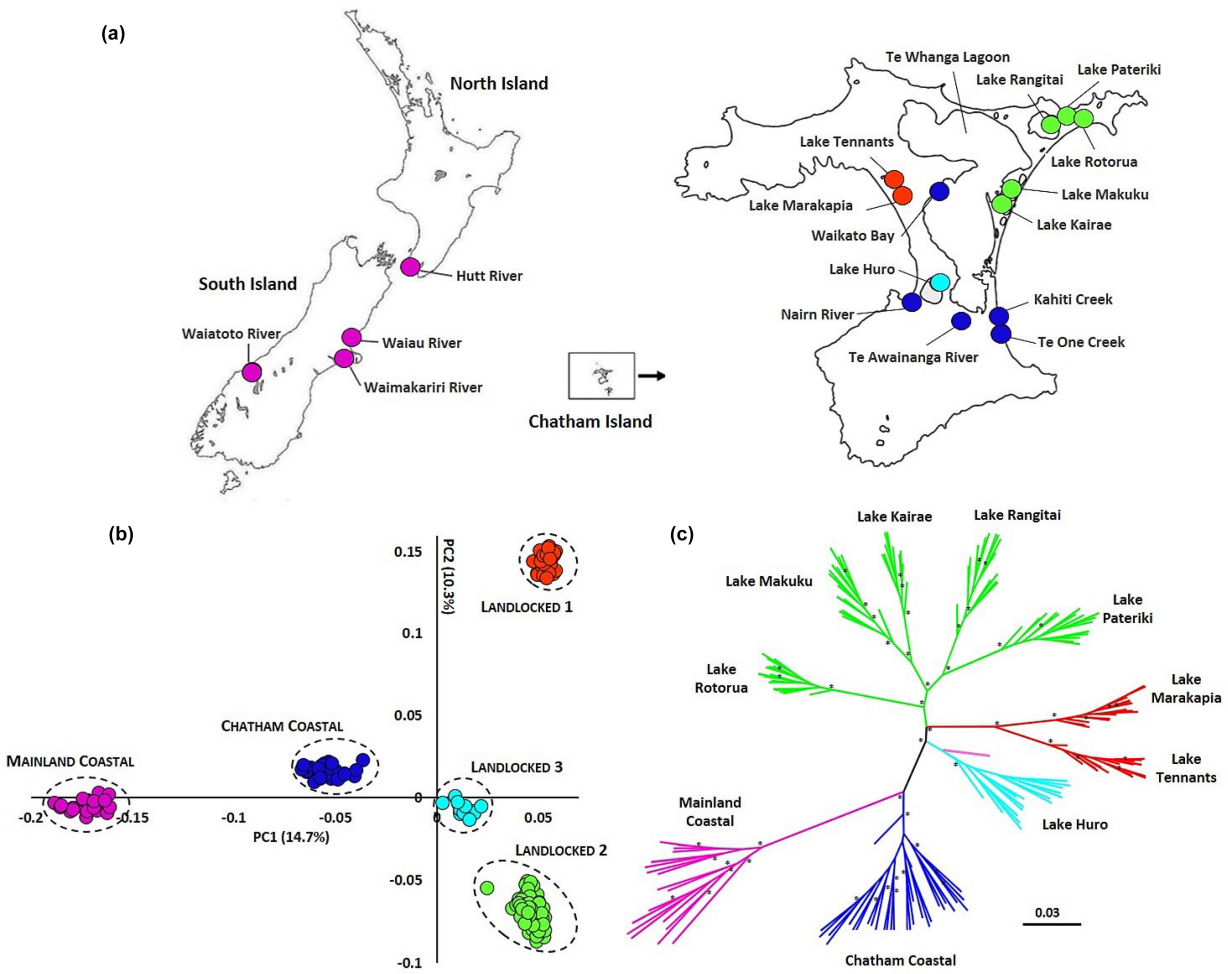
(a) Map showing the sampling locations of *Retropinna retropinna* across mainland New Zealand and Chatham Island. (b) Principal Component Analysis, based on 21,135 SNP markers, illustrating the genomic divergence among different populations of *Retropinna retropinna* from Chatham Island and mainland New Zealand. (c) Maximum likelihood phylogeny of *Retropinna retropinna* across mainland New Zealand and Chatham Island. Branch support was calculated using 1000 bootstrap replicates, with an asterisk indicating branch support values exceeding 95.

Geologically young and isolated islands present excellent opportunities to investigate the evolutionary genetics of dispersal and landlocking in diadromous species (Gonzalez‐Perez & Caujape‐Castells, 2022; Puppo et al., 2016; Saro et al., 2015). The Chatham Islands archipelago is located 800 kilometres east of the mainland (South Island) of New Zealand and is part of the largely submerged continental fragment Zealandia (McFadgen, 1994) towards the eastern end of the Chatham Rise. Despite the age of their basement rocks (70–85 million years old), the Chatham Islands only re‐emerged through tectonic uplift in the past three million years, and the land area was reduced significantly between 0.32 and 1.4 million years ago due to high interglacial sea levels (Campbell et al., 1988; Landis et al., 2008; McCulloch & Waters, 2019; Mitchell et al., 2014). This geological history suggests that freshwater species such as smelt arrived within the past three million years via long‐distance dispersal. The presence of numerous small lakes as well as Te Whanga, a large central intermittently closed and open lagoon (Figure 1a), makes Chatham Island smelt an intriguing and dynamic system in which to investigate landlocking.

The evolutionary history of landlocking in New Zealand smelt is largely unknown, though some genetic differentiation between diadromous and lacustrine (lake dwelling) smelts in the Waikato basin on the North Island was documented based on four polymorphic enzymes (Mitchell et al., 1993; Ward et al., 2005). The lacustrine Chatham smelt was originally described as a distinct species (*Retropinna chathamensis*) based on some meristic variation (McDowall, 1970; Stokell, 1949), but this species was later synonymised with *R. retropinna* because the morphological variation was thought to be a result of ecological differences in their habitats (Bonnett, 1992; McDowall, 1972). However, the distinctiveness of the landlocked Chatham smelt has not been assessed with genetic data. Furthermore, the number of independent landlocking events on Chatham Island has not been evaluated, and whether these events pre‐date human arrival on Chatham Island (between 1000 and 1500 C.E.) is unknown.

In this study, we used a genotyping‐by‐sequencing (GBS) approach to assess population genomic structure among landlocked and diadromous common smelt populations from Chatham Island and mainland New Zealand. Because of the expected restriction or elimination of gene flow among the landlocked smelt populations, we hypothesised that landlocked smelt would display a higher degree of genetic structuring among populations, and lower genetic diversity within populations than diadromous smelt. We conducted a demographic analysis to infer the history of landlocking, and specifically to assess the number and approximate age of landlocking events on Chatham Island.

## 2 MATERIALS AND METHODS

### 2.1 Sampling methods

A total of 169 common smelt were collected from 17 different lakes, rivers, streams and lagoons situated on the ‘mainland’ (North and South Islands) of New Zealand and on Chatham Island (Figure 1a, Table S1). All the Chatham Island (*N* = 151) and mainland New Zealand samples (*N* = 18) were captured using fyke and/or seine nets. Fish were immediately euthanized by immersion in a 175 mg/L solution of AQUI‐S isoeugenol (AQUI‐S New Zealand Ltd., Lower Hutt, NZ) for 5 min after collection. Pectoral fin tissue samples were then collected and preserved in absolute ethanol in the field. All protocols including live animals were approved by the University of Otago Animal Ethics Committee (AUP‐18‐200). Sites were initially categorised into five population types. Chatham Island coastal stream sites were categorised as ‘Chatham Coastal’, sites in Te Whanga Lagoon and its tributaries as ‘Te Whanga’, and Chatham Lake sites as ‘Landlocked’. Samples from the mainland were categorised as ‘North Island’ (Hutt River) and ‘South Island’ (Waiau and Waimakariri Rivers on the east coast and Waiatoto River on the west coast; Table S1).

### 2.2 DNA extraction and sequencing

Genomic DNA was extracted from the fin tissue using the Qiagen DNeasy Blood and Tissue Kit following the manufacturer's protocols. DNA concentration was quantified using a DeNovix DS‐11 Spectrophotometer (GCbiotech) and a subset of random samples was also quantified using a Qubit 2.0 fluorometer (Thermo Fisher Scientific) to check the DNA quality. Samples with DNA concentration ~25 ng/μL with a total volume of 30 μL were transferred to plates and sent to AgResearch Ltd (Invermay, Dunedin, New Zealand) for GBS library preparation. The GBS library (Lu et al., 2013) was prepared using the restriction enzyme *PstI*‐*MspI* double digest and included negative control samples (no DNA). Libraries underwent a Pippin Prep (Sage Science) to select fragments in the size range of 193–318 bp (genomic sequence plus 123 bp of adapters). Single‐end sequencing (1× 101 bp) was performed on an Illumina HiSeq2500 utilising v4 chemistry.

### 2.3 Bioinformatics and genotyping

Raw sequencing files were quality checked using FastQC v0.10.1 (Andrews, 2010). Adapters were removed and all reads were truncated to a common 65 bp length using cutadapt v.2.3 (Martin, 2011). SNP calling was performed using Stacks v2.53. The detailed SNP calling protocol is outlined in Appendix S1. Raw reads were demultiplexed according to the barcodes used for each sample using the process_radtags algorithm executed in Stacks. In the absence of a reference genome, the denovo_map.pl pipeline of Stacks was run with the default parameters. We retained SNPs that were genotyped in at least 80% of samples and removed any loci containing SNPs with heterozygosity above 65%, as these loci are potentially problematic merged paralogs. These filtering steps resulted in the retention of 21,135 SNPs for 169 individuals.

### 2.4 Population genomic structure analysis

Population structure across the entire dataset was assessed with principal component analysis (PCA) using the package pcadapt v.4.3.3 in the R environment (Luu et al., 2017; R Core Team, 2020). Furthermore, we used fastSTRUCTURE to estimate the number of genetic clusters in the data set (Raj et al., 2014). For this analysis, the vcf file was converted to fastStructure input files using PGDSpider v.2.1.1.5 (Lischer & Excoffier, 2012). Putative genetic clusters (K) from K = 2 to K = 17 were run, as there were 17 sampling sites left after the final SNP calling. The value of K that maximised the marginal likelihood was taken as optimal. R packages pophelper v.2.3.1 (Francis, 2017) and ggplot2 v.3.3.5 (Wickham, 2009) were used for visualisation. The R packages adegenet v.2.1.3 (Jombart, 2008) and hierfstat v.0.5‐7 (Goudet, 2005) were used to calculate overall population genomic statistics including observed heterozygosity (*H*_O_), expected heterozygosity (*H*_S_), overall gene diversity (*H*_T_), amount of gene diversity among samples (*D*_ST_), fixation index (*F*_ST_), inbreeding coefficient (*F*_IS_) and population differentiation (*D*_EST_). Pairwise *F*_ST_ values were also calculated among population types utilising 100 bootstraps.

### 2.5 Phylogenetic analysis

For phylogenetic analysis, the SNP dataset was converted to phylip format using the script *vcf2phylip.py* (Ortiz, 2019). Phylogenomic relationships among populations were assessed using Maximum Likelihood in IQ‐Tree v2.1.2 (Minh et al., 2020; Nguyen et al., 2014). We used a generalised time‐reversible model that accounted for among‐site rate variation (GTR + G), with 1000 bootstraps. Phylogenetic trees were visualised in FigTree v.1.4.4 (http://tree.bio.ed.ac.uk/software/figtree/).

### 2.6 Demographic history analysis

To infer the history of landlocking we conducted coalescent analysis using approximate Bayesian computation (ABC) implemented in DIYABC Random Forest version 1.0.14 (Collin et al., 2021). Based on the clustering of samples evident from the PCA, we placed the samples into five groups for demographic history analysis: three groups of Chatham Island landlocked populations (CILL1, CILL2 and CILL3), Chatham Island Coastal fish including coastal and Te Whanga sites (CIC) and Mainland Coastal including North and South Island samples (MC) (Table S2).

**FIGURE 2:**
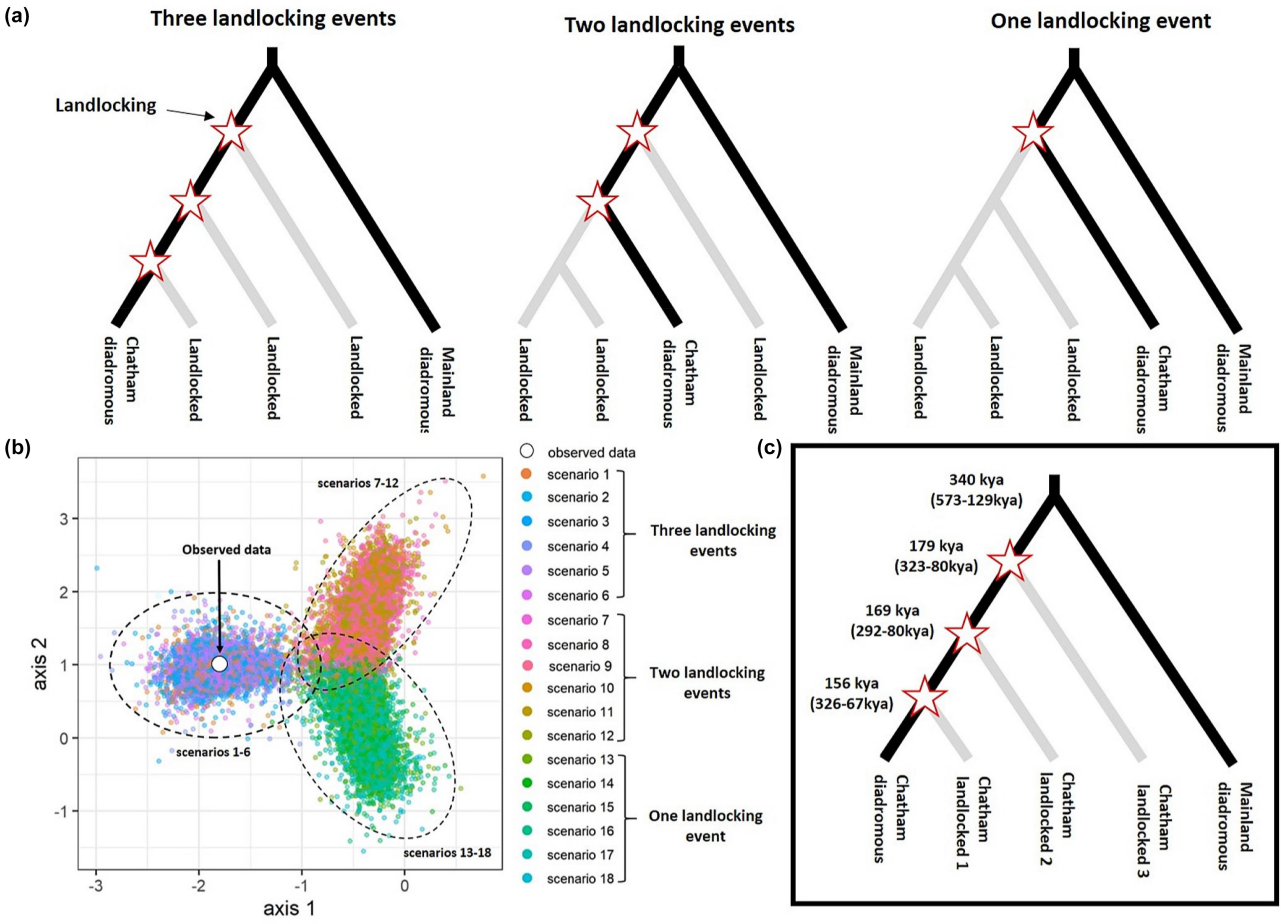
Coalescent analyses support repeated independent landlocking of *Retropinna retropinna* on Chatham Island. (a) Outline of the three potential evolutionary histories of the landlocked *R. retropinna* populations on Chatham Island. See Figure S1 for a complete list of all demographic scenarios compared in DIYABC‐RF. (b) Principal Component Analysis evaluating the fit between observed summary statistics and simulated datasets for 18 demographic scenarios of *R. retropinna* on Chatham Island. This analysis confirms that the observed data were contained within the space of the posterior distribution of the summary statistics for best model scenarios (three landlocking events). (c) The most well‐supported scenario for the evolution of landlocked *R. retropinna* populations (scenario 1; three landlocking events; see Figure S1).

We compared 18 demographic scenarios to determine the most likely landlocking history of *R. retropinna* on Chatham Island (Figure S1). We assumed that the ancestor that migrated to Chatham Island was a diadromous smelt, and therefore that MC‐CIC was the earliest divergence. Although the scenarios do not account for the history of every individual landlocked population, the PCA demonstrated that the three clusters account for most of the genetic variation in landlocked smelt, and we needed a manageable number of biologically realistic scenarios. Scenarios 1–6 represented three independent landlocking events where Chatham Island's landlocked populations (CILL1, CILL2 and CILL3) sequentially diverged from their common ancestral Chatham diadromous populations (CIC) in some order (Figure 2a, Figure S1a). Scenarios 7–12 included two landlocking events (Figure 2a, Figure S1b), while scenarios 13–18 represent a single landlocking event, followed by divergence among the landlocked populations (Figure 2a, Figure S1c).

SNPs that were not found in at least one sample were removed, as were SNPs with Minor Allele Frequency (MAF) <0.05. The vcf file was converted to DIYABC input format using the vcf2DIYABC.py script (https://github.com/loire/vcf2DIYABC.py) in Python version 2.5 (van Rossum & Drake, 2001). As population sizes and divergence timeframes are unknown, we used broad priors with a uniform distribution (10^1^–10^6^ for each parameter). After generating the training set simulation, the ‘Random Forest analyses’ module of DIYABC was used to conduct model scenario choice and parameter estimation in DIYABC‐RF.

In scenario choice prediction, DIYABC returns votes for each scenario which indicates the number of times a scenario is picked in a forest of *n* trees. In this case, the scenario with the most classification votes among the examined scenarios is the one that is the best suited to the target dataset. The outputs also include an estimate of the best‐supported scenario's posterior probability. So, for each scenario, 20,000 simulations and 500 trees were run to identify the model scenario with the most support. Before that, the prior scenario checking pre‐analysis was done to uncover potential model misspecification in the scenarios using a PCA comparing the prior and posterior parameter distributions. 1,000,000 simulations per scenario for parameter estimation were also performed. This approach estimates the population sizes and divergence times among lineages under a given scenario in the context of logistic regression. The population age or divergence timing was estimated based on one generations per year smelt lifecycle. In addition, the proportion of global (prior) error and local (posterior) error in the best‐supported model was calculated to assess the robustness of inference using 1000 pseudo‐observed data sets.

## 3 RESULTS

### 3.1 Population genomic structure

A total of 273,915,021 reads were obtained from genotyping by sequencing and after the quality filtering and trimming, 125,989,758 reads were retained. The average number of reads per sample ranged from 201,585 to 1,312,470 (mean = 714,599). SNP calling yielded 21,135 SNPs for 169 individuals (median depth per loci: 16.3; min: 6.7; max: 29.8).

We found the highest values of observed (*H*_O_) and expected heterozygosity (*H*_S_) in mainland coastal populations (South Island: *H*_O_ = 0.1267 and *H*_S_ = 0.1390; North Island: *H*_O_ = 0.1121 and *H*_S_ = 0.1294). Chatham coastal and Te Whanga populations had medium values of *H*_O_ (0.1013 and 0.0950) and *H*_S_ (0.1005 and 0.0994), while landlocked populations had lower values of *H*_O_ (0.0693) and *H*_S_ (0.0709) (Table 1). Furthermore, we found higher genetic diversity (*H*_T_) in all diadromous populations compared to landlocked populations. Specifically, the highest *H*_T_ values were observed in South Island populations (*H*_T_ = 0.1419), followed by North Island (*H*_T_ = 0.1294), Chatham coastal (*H*_T_ = 0.1016), Te Whanga (*H*_T_ = 0.0992) and landlocked populations (*H*_T_ = 0.0942) (Table 1). Highly significant pairwise *F*_ST_ values were found among population groups, except between Chatham coastal and Te Whanga populations (*F*_ST_ = 0.003), and between North and South Islands populations (*F*_ST_ = 0.043). Pairwise genetic differences were high between landlocked and South Island (*F*_ST_ = 0.296) and between landlocked and North Island (*F*_ST_ = 0.276) coastal populations, and medium‐high between landlocked and Chatham coastal (*F*_ST_ = 0.138) and between landlocked and Te Whanga (*F*_ST_ = 0.136) populations. Highly significant genetic differences were also found between Chatham and mainland diadromous populations (Table 2).

**TABLE 1:** Overall population genomic statistics for *Retropinnna retropinna* based on 21,135 SNP loci.

	*H*_O_	*H*_S_	*H*_T_	*D*_ST_	*F*_ST_	*F*_IS_	*D*_EST_
Landlocked	0.0693	0.0709	0.0942	0.0234	0.2480	0.0218	0.0288
Chatham Coastal	0.1013	0.1005	0.1016	0.0011	0.0107	−0.0076	0.0018
Te Whanga	0.0950	0.0994	0.0992	−0.0002	−0.0015	0.0442	−0.0003
South Island	0.1267	0.1390	0.1419	0.0029	0.0205	0.0887	0.0051
North Island	0.1121	0.1294	0.1294	0	0	0.1335	–

Abbreviations: *D*_EST_, a measure of population differentiation; *D*_ST_, amount of gene diversity among samples; *F*_IS_, inbreeding coefficient; *F*_ST_, fixation index; *H*_O_, observed heterozygosity; *H*_*S*_, expected heterozygosity; *H*_T_, overall gene diversity.

**TABLE 2:** Pairwise *F*_ST_ values among five population groups of *Retropinna retropinna* across Chatham Island and mainland New Zealand.

	Chatham coastal	Landlocked	North Island	South Island	Te Whanga
Chatham Coastal	0				
Landlocked	**.138**	0			
North Island	**.245**	**.296**	0		
South Island	**.197**	**.276**	.043	0	
Te Whanga	.003	**.136**	**.248**	**.209**	0

*Note*: Bold values indicate significant *p*‐values.

Principal component analysis revealed a clear separation between landlocked and diadromous common smelt (Figure 1b). Mainland populations formed one cluster (MC), Chatham coastal populations including Te Whanga Lagoon formed another (CIC) and Chatham landlocked populations formed three regionally separated clusters. Among these three landlocked clusters, cluster‐1 (CILL1) represented two lakes in the western part of the Island, Lake Marakapia and Tennants Lake. Cluster‐2 (CILL2) included all sampled lakes in the northeast of Chatham Island (Lake Kairae, Lake Makuku, Lake Pateriki, Lake Rotorua and Lake Rangitai). Cluster‐3 (CILL3) only included Lake Huro (Figure 1).

**FIGURE 3:**
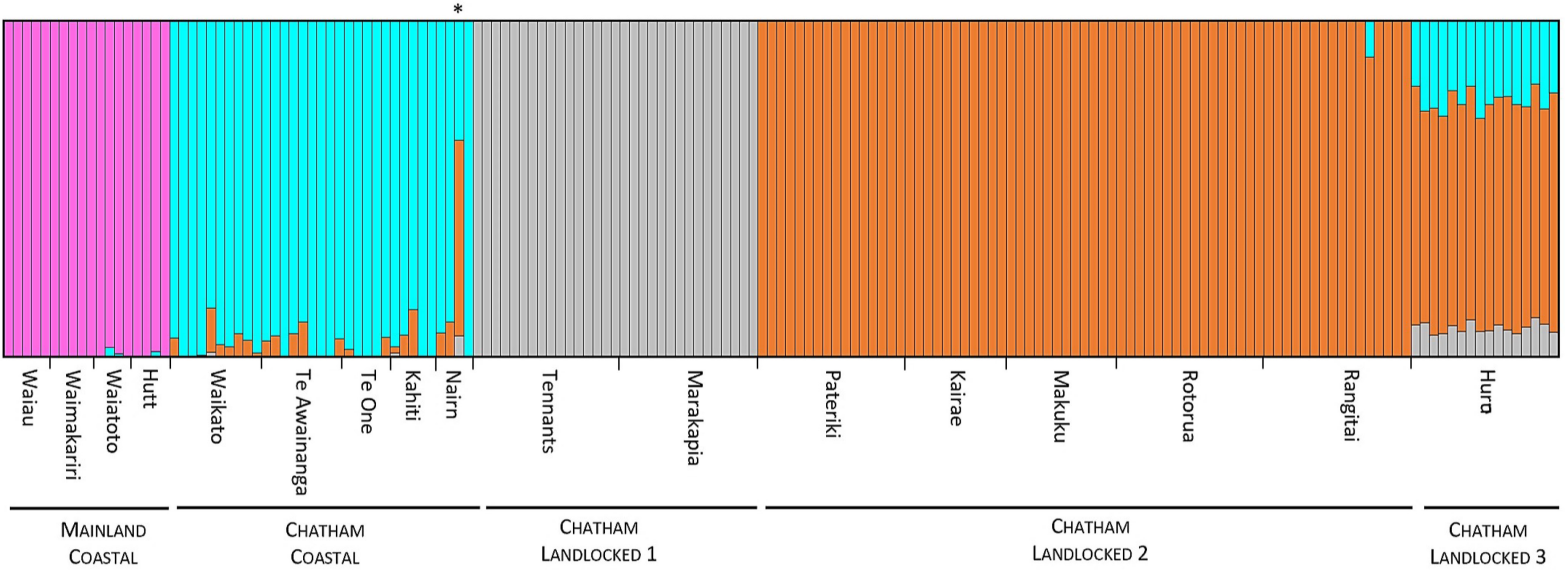
Population genomic structuring in *Retropinna retropinna* inferred in fastSTRUCTURE for K = 4 (the most likely value of K). Each vertical bar represents an individual, with the proportion of colour in each bar indicating the posterior probability of assignment of the individual to each of the four clusters. The anomalous fish from Nairn River is indicated with an asterisk.

Population genomic structure analysis using fastSTRUCTURE suggested that the optimum number of population clusters in our dataset was four. The Chatham coastal populations formed an isolated cluster that is distinct from the mainland coastal cluster (Figure 3). However, a single fish from the Nairn River clustered more closely to the Lake Huro fish than to other fish from the Nairn River. Lakes Tennants and Marakapia formed a landlocked cluster, while Lakes Pateriki, Kairae, Makuku, Rotorua and Rangitai formed another landlocked cluster without any inferred admixture. Our fastSTRUCTURE analysis suggests that the Lake Huro population may have been formed by admixture between other Chatham landlocked populations and the Chatham coastal populations (Figure 3).

### 3.2 Phylogenetic analysis

Phylogenetic analysis identified a similar pattern to the principal component analysis, with the Mainland Coastal, Chatham Coastal and three landlocked Chatham populations each forming well‐supported clades (Figure 1c). Furthermore, genetic structuring was evident within each of the landlocked Chatham populations, with samples from each lake forming a monophyletic clade. The tree topology showed a single split between Chatham landlocked populations and Chatham coastal populations, with the landlocked populations forming a monophyletic clade (Figure 1c).

### 3.3 Demographic history of Chatham landlocked populations

The best‐supported model, according to the demographic analysis, was scenario 1, which includes three independent landlocking events (Figure 2; Table S3). The next highest supported scenarios also include three landlocking events (Table S3). In contrast, all one‐ and two‐landlocking scenarios had fewer classification votes than any of the three‐landlocking scenarios (Table S3). The best‐supported scenario has Lake Huro as the first to diverge from the ancestral Chatham diadromous populations (ca. 179,163 generations ago, 95% CI = 80,349–323,468) followed by landlocked cluster 2 (ca. 169,467 generations ago, 95% CI = 80,182–292,275) and landlocked cluster 1 (ca. 156,273 generations ago, 95% CI = 67,259–326,997), respectively (Figure 2c; Table S4). The PCA of the 18 demographic scenarios confirmed that the observed data were contained within the space of the posterior distribution of the summary statistics for best model scenarios of three landlocking events (Figure 2b). We also found that overall prediction errors were low (0.83), and the estimated proportions of prior errors (0.18 for CIC, 0.31 for CILL1, 0.23 for CILL2, 0.19 for CILL3 and 0.29 for MC) and posterior errors (0.45 for CIC, 0.25 for CILL1, 0.28 for CILL2, 0.28 for CILL3 and 0.43 for MC) were relatively low for the best‐supported scenario (scenario 1).

## 4 DISCUSSION

Our genome‐wide analysis revealed that the Chatham Island populations of common smelt, *R. retropinna*, are genetically distinct from populations on New Zealand's mainland. Furthermore, our analyses indicated that the Chatham landlocked populations form a monophyletic clade that is significantly genetically diverged from diadromous populations. Consistent with expectations, diadromous populations had notably higher genetic diversity than landlocked populations. We also identified significant population structuring among several landlocked populations on Chatham Island. Intriguingly, our coalescent analyses support three independent landlocking events on Chatham Island, with this loss of diadromy significantly pre‐dating human colonisation on Chatham Island.

### 4.1 Genomic structure of Chatham landlocked smelt populations

Population genomic analyses identified five distinct common smelt populations across Chatham Island and mainland New Zealand. The South and North Island coastal populations formed a single genetic cluster with no significant population genetic structuring evident. While we lacked coverage for some regions of mainland New Zealand, with the only North Island population (Hutt River) located at the very southern tip of the North Island, gene flow and migration among the mainland does seem sufficient to prevent genomic differentiation. Chatham coastal populations formed their own cluster that included Te Whanga populations, indicating extensive migration and gene flow between the two groups. While Te Whanga is periodically isolated from the ocean, connectivity during periods of lagoon opening appears to be sufficient to homogenise these populations. Pairwise F_*ST*_ also showed population genomic differences between Chatham and mainland coastal smelt that suggests little ongoing migration to or from Chatham Island.

For the Chatham landlocked common smelt populations, three clear genetic clusters were found. Lake Marakapia and Tennants Lake in the western part of Chatham Island formed one landlocked cluster; Lakes Kairae, Makuku, Pateriki, Rotorua and Rangitai in the north‐east formed another landlocked cluster; and Lake Huro formed a third cluster on its own. STRUCTURE identified Lake Huro as a potentially admixed population with ancestry including Chatham coastal and landlocked clusters. Lake Huro is the one lake with a current connection with the sea, with a small outlet stream draining into the Nairn River. The finding of a single fish in the Nairn River that genetically clustered with Lake Huro confirms that migration does occur, but this must be at a low enough level that genetic mixing is limited. Lake Huro smelt displays body shape changes comparable to other Chatham landlocked populations (M. G. Ara, unpublished data), further supporting their identity as a landlocked population that experiences at most occasional migration. The other two landlocked clusters showed some genetic differentiation among lakes, although the majority of the genetic variation was accounted for by differences between clusters. Similar to our findings, high‐genetic divergence has been found between diadromous and freshwater resident populations of Arctic char, *Salvelinus alpinus* (Salisbury et al., 2018), Atlantic salmon, *Salmo salar* (Perrier et al., 2013; Tonteri et al., 2007), threespine stickleback, *Gasterosteus aculeatus* (Drevecky et al., 2013; Reusch et al., 2001; Takamura & Mori, 2005), and rainbow trout, *Oncorhynchus mykiss* (Bowersox et al., 2016).

### 4.2 History of smelt colonisation and landlocking on Chatham Island

Our coalescent demographic analyses suggest that the Chatham diadromous smelt populations diverged from mainland New Zealand diadromous smelt populations around 0.34 million years ago (95% CI = 0.13–0.57), assuming a typical one‐year generation time. We did not have suitable fossil or biogeographic calibration points to estimate divergence time based on substitution rates, and the estimates from the demographic analysis are relatively imprecise. Nonetheless, the estimates still clearly place smelt arrival on Chatham Island well after the island emergence ca. 3 million years ago, consistent with molecular studies in a range of other taxa (Arensburger et al., 2004; Campbell et al., 2009; McCulloch & Waters, 2019; McGaughran et al., 2006; Miskelly, 2008; Trewick, 2000). The estimated times of colonisation and landlocking and the deep Pleistocene divergence among genetic clusters also effectively rule out human translocation as the primary means of landlocking. Moriori, the first human settlers of the Chatham Islands, arrived between 1000 and 1500 C.E. (Dodson & Kirk, 1978; McFadgen, 1994; Richards, 1952, 1972), and common smelt (porure) whitebait is an important traditional food source, especially in Te Whanga (McDowall, 2010). Our analysis suggests that both coastal and landlocked smelt populations were well‐established before human arrival. While some recent translocations among lakes within landlocked clusters are still possible, the presence of smelt in Chatham lakes does not appear to be the result of human intervention, unlike documented cases of smelt introductions to some North Island lakes (Strickland, 1993).

A key factor in studying the genetic and phenotypic consequences of landlocking is whether populations have become landlocked independently, or formed via the dispersal of a single landlocked ancestor. The phylogenetic tree topology included a monophyletic landlocked group, implying that they shared a landlocked ancestor. However, this interpretation is complicated by the fact that there is a single well‐mixed diadromous population that has continued to evolve and possibly receive colonists from the mainland following landlocking. The extensive gene flow within all Chatham diadromous smelt has the potential to obscure any historical relationships between landlocked clades and specific ancestral populations. We used a more versatile demographic analysis to infer support for alternative population histories. The best‐supported scenario indicated that the Lake Huro landlocked population diverged earliest from the ancestral diadromous population, followed by landlocked cluster 2 (Lake Pateriki, Lake Kairae, Lake Makuku, Lake Rotorua and Lake Rangitai) and then landlocked cluster 1 (Tennants Lake and Lake Marakapia). The specific order of divergence was not strongly supported, but all of the best‐supported scenarios included three landlocking events. The coalescent analysis thus suggests that at least three landlocking events have occurred on Chatham Island's common smelt populations. It is also possible that some of the lakes within each cluster also became landlocked independently – for example, Lake Pateriki occasionally connects via a sand bar to the north coast, whilst the other lakes in the northeast cluster would more likely have been colonised via Te Whanga Lagoon. We also did not include every lake containing smelt populations in our GBS sampling, so we regard three landlocking events on Chatham Island as a minimum.

Several mechanisms, including postglacial rebound, physical impoundment, land use change and man‐made barriers have led diadromous fish to become isolated in lakes and other freshwater habitats after post‐glacial colonisation (Lee & Bell, 1999; Waters et al., 2020; Zemanova & Ramp, 2021). It is unknown what specific landscape changes led to *R. retropinna* landlocking on Chatham Island. Sea level has fluctuated in parallel with historic climate, with the sea level being around 135–150 m lower during glacial periods of the Pleistocene. The large central Te Whanga Lagoon is a dominant feature of Chatham Island. While Te Whanga is currently brackish and frequently open to the ocean, it has gone through periods of being more freshwater, likely including these times of low sea level. Te Whanga Lagoon developed at the present sea level (during the current 10,000‐year interglacial) by the creation of barrier beaches between volcanic and limestone land that remained above the sea (McFadgen, 1994). During the Pleistocene period, it is plausible that common smelt adapted to freshwater residence within Te Whanga, and subsequently colonised different lakes during periods of flooding. Alternatively, it is possible that some lakes, especially Lake Huro, were directly colonised by the ocean during times of higher sea levels.

### 4.3 Genetic diversity of Chatham landlocked smelt populations

Diadromous *R. retropinna* populations in our study were more genetically diverse and had higher heterogeneity than landlocked populations. As landlocked smelt populations became isolated from migratory populations by natural barriers, they showed lower genetic diversity likely due to reduced gene flow (Bowersox et al., 2016; DeWoody & Avise, 2000). This genetic diversity could also be attributed to founder effects and increased genetic drift during their freshwater colonisation (Deagle et al., 2013; Jones et al., 2012; Perrier et al., 2013; Willoughby et al., 2018). Low and fluctuating population size may also have contributed to the loss of genetic diversity in freshwater‐isolated populations (Perrier et al., 2013), though there is no direct information on landlocked and diadromous population sizes of Chatham smelt. Similar to our study, lower levels of genetic diversity have also been observed in landlocked populations of freshwater Atlantic salmon (*Salmo salar*) (Bourret et al., 2013; Perrier et al., 2013; Tonteri et al., 2007), common galaxias (*Galaxias maculatus*) (Delgado et al., 2019), ide (*Leuciscus idus*) (Skovrind et al., 2016), perch (*Perca fluviatilis*) (Olsson et al., 2011), rainbow trout (*Oncorhynchus mykiss*) (Bowersox et al., 2016; Narum et al., 2008) and three‐spine stickleback (*Gasterosteus aculeatus*) (Drevecky et al., 2013; Ferchaud & Hansen, 2016) compared with their migratory counterparts. Our genomic analysis also confirmed that there is a significant population differentiation between Chatham landlocked and diadromous common smelt as well as among landlocked populations. In contrast, no significant population differentiation was observed among Chatham coastal populations including Te Whanga, or among mainland coastal populations, indicating ongoing or recent gene flow.

To summarise, ancestrally diadromous smelt appears to have been landlocked multiple times in different lakes on Chatham Island. Our results illustrate how landlocking of diadromous fish can occur repeatedly, across very narrow spatial scales. The distinct environment of lakes may lead to adaptive evolution in landlocked populations, which will be the subject of future research. Although preliminary results indicate that landlocked and diadromous *R. retropinna* populations on Chatham Island differ in morphological traits including body shape (M. G. Ara, unpublished data), we do not yet know to what degree phenotypic shifts represent genetic change versus phenotypic plasticity. The presence of replicated landlocking on an isolated island presents an outstanding natural laboratory to investigate the genomic basis of adaptation following the landlocking of normally diadromous fish.

## AUTHOR CONTRIBUTIONS

**Motia G. Ara:** Conceptualization (lead); formal analysis (lead); methodology (lead); software (equal); writing – original draft (lead). **Graham A. McCulloch:** Formal analysis (supporting); methodology (supporting); software (equal); writing – review and editing (supporting). **Ludovic Dutoit:** Formal analysis (supporting); methodology (supporting); software (equal); writing – review and editing (supporting). **Graham P. Wallis:** Supervision (supporting); writing – review and editing (supporting). **Travis Ingram:** Conceptualization (equal); formal analysis (supporting); methodology (equal); software (equal); supervision (lead); writing – review and editing (lead).

## CONFLICT OF INTEREST STATEMENT

The authors declare that they have no competing interests.

## Supporting information


Figure S1.

Appendix S1.

## Data Availability

All sequencing data used in this study can be found (demultiplexed by sample) on the NCBI Sequence Read Archive PRJNA1054659.
