# Supplementary figures and images for: Genomics reveals repeated landlocking of diadromous fish on an isolated island
Source: Ecol Evol. 2024 Feb 16;14(2):e10987. doi: 10.1002/ece3.10987 (PMC10870334; doi:10.1002/ece3.10987)

(a)

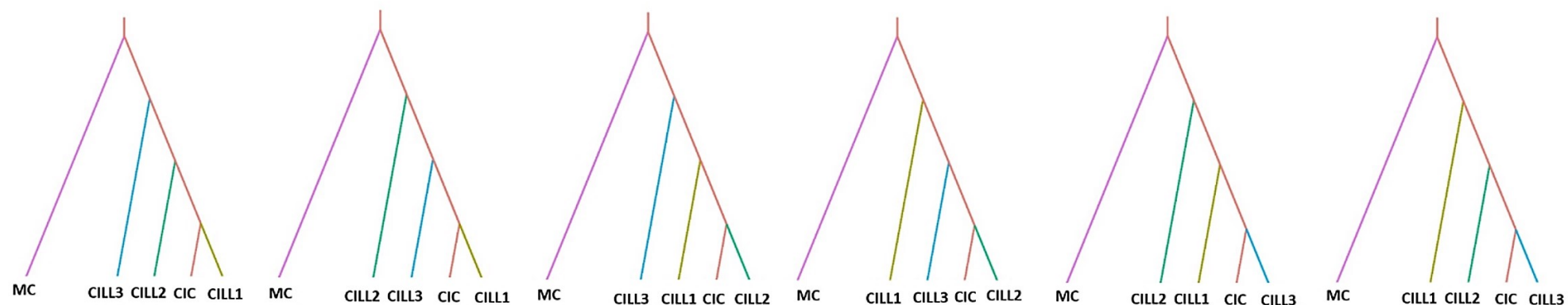

(b)

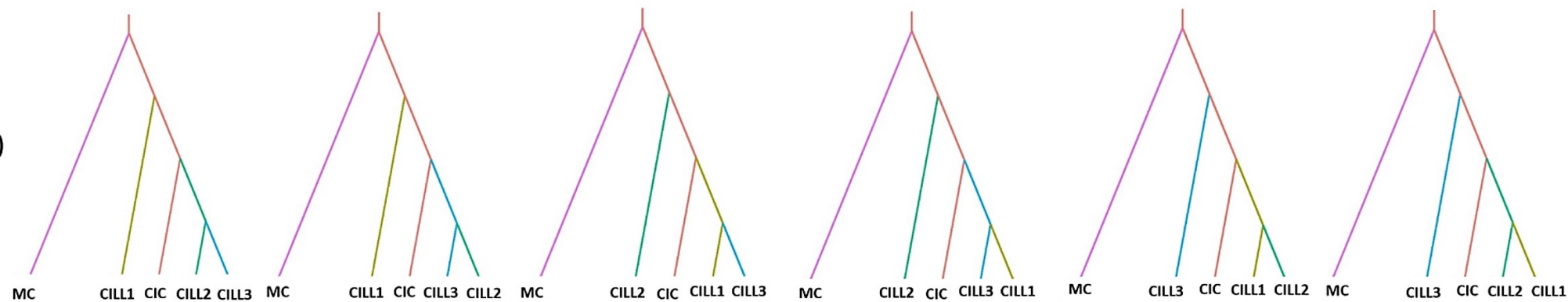

(c)

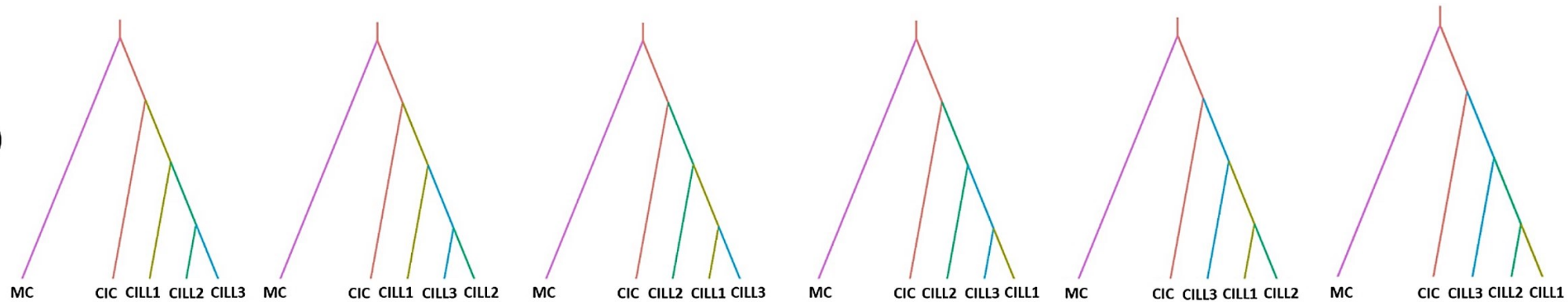

Supplement: Supplementary file 1 — Figure S1. [file ECE3-14-e10987-s002.pdf]
