# Supplementary material for: Genomics reveals repeated landlocking of diadromous fish on an isolated island
Source: Ecol Evol. 2024 Feb 16;14(2):e10987. doi: 10.1002/ece3.10987 (PMC10870334; doi:10.1002/ece3.10987)
Supplement: Supplementary file 2 — Appendix S1. [file ECE3-14-e10987-s001.pdf]

Supplementary Tables

Table S1 Sampling details of Common smelt, *Retropinna retropinna,* across Chatham Island and mainland New Zealand

Sampling category	Locality	Coordinates	Samples
Landlocked	Lake Tennants	-43.82807, -176.56806	16
	Lake Pateriki	-43.93544, -176.50076	16
	Lake Huro	-43.93544, -176.50076	16
	Lake Marakapia	-43.84615, -176.55055	15
	Lake Kairae	-43.86045, -176.39740	10
	Lake Makuku	-43.84586, -176.39029	14
	Lake Rotorua	-43.75745, -176.29078	16
	Lake Rangitai	-43.77077, -176.32150	15
Te Whanga	Waikato Bay	-43.86045, -176.39740	10
	Te Awainanga River	-43.98880, -176.45269	9
Chatham Coastal	Te One Creek	-44.01978, -176.38270	5
	Kahiti Creek	-43.99916, -176.40193	5
	Nairn River	-43.95131, -176.55654	4
South Island	Waiau River	-42.72677,-173.23928	5
	Waimakariri River	-43.41560,-172.64458	5
	Waiatoto River	-43.98266, 168.79230	4
North Island	Hutt River	-41.16281, 174.97469	4

Table S2 Details of different population clusters used to test the history of landlocking in *Retropinna retropinna* on Chatham Island with DIYABC-RF analysis

**Clusters**	**Abbreviation**	**Sites included**
Chatham Landlocked 1	CILL1	Lake Tennants
		Lake Marakapia
Chatham Landlocked 2	CILL2	Lake Pateriki
		Lake Kairae
		Lake Makuku
		Lake Rotorua
		Lake Rangitai
Chatham Landlocked 3	CILL3	Lake Huro
Chatham Island Coastal	CIC	Waikato Bay
		Te Awainanga River
		Te One Creek
		Kahiti Creek
		Nairn River
Mainland Coastal	MC	Waiau River
		Waimakariri River
		Waiatoto River
		Hutt River

Table S3 Results of DIYABC-RF coalescent analysis assessing the demographic history of *Retropinna retropinna* on Chatham Island. PP = Posterior Probability.

	**Three landlocking events**						**Two landlocking events**						**Single landlocking event**					
**Model**	**1**	**2**	**3**	**4**	**5**	**6**	**7**	**8**	**9**	**10**	**11**	**12**	**13**	**14**	**15**	**16**	**17**	**18**
**Votes**	**100**	59	87	60	89	49	4	8	6	6	6	4	3	2	3	3	8	3
**PP**	**0.45**	0.26	0.39	0.27	0.40	0.22	0.02	0.04	0.03	0.03	0.03	0.02	0.01	0.01	0.01	0.01	0.04	0.01

Table S4 Posterior distributions of population parameters for the favoured demographic scenario for the evolutionary history of *Retropinna retropinna* on Chatham Island (scenario 1; see Table S3). Effective population size (*N*e) of Chatham coastal (CIC), Chatham landlocked group 1 (CILL1), Chatham landlocked group 2 (CILL2), Chatham landlocked group 3 (CILL3), and mainland (MC). Time of divergence (in generations) between CIC and CILL1 populations (t1), between CIC and CILL2 populations (t2), between CIC and CILL3 populations (t3), and between CIC and MC populations (t4).

Parameter	Expectation	Median	Quantile_0.05	Quantile_0.95	Variance
***N*e (CIC)**	376383	346847	133211	757140	2.15806e+10
***N*e (CILL1)**	712230	738734	354125	979689	2.77518e+10
***N*e (CILL2)**	666565	687964	312135	971506	2.81119e+10
***N*e (CILL3)**	706322	734018	332695	977618	3.2524e+10
***N*e (MC)**	634198	619230	297355	957755	5.22825e+10
**t1**	164652	156273	67259.4	326997	4.56218e+09
**t2**	175891	169467	80182	292275	2.8261e+09
**t3**	190423	179163	80349.4	323468	3.75539e+09
**t4**	338963	340241	129171	573364	1.83262e+10
